# Measles Virus Host Invasion and Pathogenesis

**DOI:** 10.3390/v8080210

**Published:** 2016-07-28

**Authors:** Brigitta M. Laksono, Rory D. de Vries, Stephen McQuaid, W. Paul Duprex, Rik L. de Swart

**Affiliations:** 1 Department of Viroscience, Erasmus MC, 3015CN Rotterdam, The Netherlands; b.laksono@erasmusmc.nl (B.M.L.); r.d.devries@erasmusmc.nl (R.D.d.V.); 2 Centre for Cancer Research and Cell Biology, Queen’s University of Belfast, BT7 1NN Belfast, UK; s.mcquaid@qub.ac.uk; 3 Department of Microbiology, Boston University School of Medicine, Boston, MA 02118, USA; pduprex@bu.edu

**Keywords:** measles virus, immune suppression, pathogenesis, tropism, transmission

## Abstract

Measles virus is a highly contagious negative strand RNA virus that is transmitted via the respiratory route and causes systemic disease in previously unexposed humans and non-human primates. Measles is characterised by fever and skin rash and usually associated with cough, coryza and conjunctivitis. A hallmark of measles is the transient immune suppression, leading to increased susceptibility to opportunistic infections. At the same time, the disease is paradoxically associated with induction of a robust virus-specific immune response, resulting in lifelong immunity to measles. Identification of CD150 and nectin-4 as cellular receptors for measles virus has led to new perspectives on tropism and pathogenesis. In vivo studies in non-human primates have shown that the virus initially infects CD150^+^ lymphocytes and dendritic cells, both in circulation and in lymphoid tissues, followed by virus transmission to nectin-4 expressing epithelial cells. The abilities of the virus to cause systemic infection, to transmit to numerous new hosts via droplets or aerosols and to suppress the host immune response for several months or even years after infection make measles a remarkable disease. This review briefly highlights current topics in studies of measles virus host invasion and pathogenesis.

## 1. Introduction

Measles virus (MV) is the prototype member of the genus *Morbillivirus,* the subfamily *Paramyxovirinae* and the family *Paramyxoviridae*. MV is an enveloped virus with a single strand, non-segmented negative sense RNA genome and exclusively causes disease in old- and new-world non-human primates (NHPs) and humans. Like all morbilliviruses, MV is highly contagious and is transmitted via the respiratory route [1]. Once the virus is inhaled and a primary target cell is infected, systemic spread ensues and clinical signs appear after 9–19 days. The prodromal stage starts with fever and malaise associated with cough, coryza and conjunctivitis, colloquially the three “C’s”. During this stage Koplik’s spots can be observed on the buccal mucosa. On the subsequent days patients develop a maculopapular skin rash that starts behind the ears and spreads to the face, trunk and extremities [2,3]. MV infection is usually self-limiting, due to the clearance of virus-infected cells by the immune system. Recovery is followed by lifelong immunity to measles. In rare cases, severe measles-associated central nervous system (CNS) complications may develop: Acute disseminated encephalomyelitis (ADEM), measles inclusion body encephalitis (MIBE) or subacute sclerosing panencephalitis (SSPE). MV infection paradoxically also results in a transient immune suppression that may last over two years after infection and leads to opportunistic infections and increased mortality risk [4]. The World Health Organisation (WHO) estimated that approximately 114,900 people, mostly children under five years of age, died of measles and resulting sequelae in 2014 [5].

Signalling lymphocyte activation molecule family member 1 (SLAMF1, also known as CD150), which is expressed by subsets of thymocytes, dendritic cells (DCs), haematopoietic stem cells (HSCs), macrophages, T- and B-cells, has been identified as a cellular receptor for MV [2,6,7]. Infection of NHPs with recombinant MV (rMV) derived from the wild-type Japanese IC323 strain and engineered to express a fluorescent reporter protein identified CD150^+^ lymphocytes and DCs as predominant target cells of MV infection in vivo [8]. Nectin cell adhesion molecule 4 (nectin-4, previously also known as poliovirus receptor-related 4 or PVRL4) has been identified as another cellular receptor for MV that is expressed by epithelial cells [9,10]. This protein is part of the adherens junction complex, which is located at the basolateral side of the epithelium, underneath the tight junctions. Nectin-4 is also expressed by keratinocytes [11,12] and endothelial cells [13], suggesting a potential role for these cell types in the pathogenesis of the characteristic measles skin rash [13,14,15].

Both CD150 and nectin-4 play crucial roles in the pathogenesis of measles. Vaccine and laboratory-adapted MV strains can utilise CD46 as an additional cellular receptor in vitro, but this receptor does not seem to play a major role during infection with these viruses in vivo [16,17]. The C-type lectins DC-specific intercellular adhesion molecule-3-grabbing non-integrin (DC-SIGN) and Langerin, expressed by DCs and Langerhans cells, respectively, have been identified as attachment receptors for MV. However, these molecules do not mediate MV entry, but are thought to “capture” MV particles and facilitate CD150-mediated virus-to-cell fusion of DCs or lymphocytes [18,19]. The following review of MV entry, dissemination, transmission and immune suppression is largely based on observations from experimental MV infections of NHPs.

## 2. Entry

Respiratory epithelial cells have classically been considered as the early target cells of MV infection in the respiratory tract. However, the lack of CD150 or nectin-4 expression on their apical surface renders this entry mechanism unlikely. NHPs infected with rMV that was unable to recognise nectin-4 (referred to as “nectin-4-blind” virus) still led to the establishment of systemic infection [20], whereas animals infected with an rMV that was unable to recognise CD150 (“CD150-blind”) failed to develop clinical signs or viremia [21]. These findings highlight the importance of CD150 during viral entry and exclude respiratory epithelial cells as the primary target cells, although it cannot be excluded that receptor-binding modifications have led to a generalized attenuation and loss of viral fitness.

**Figure 1:**
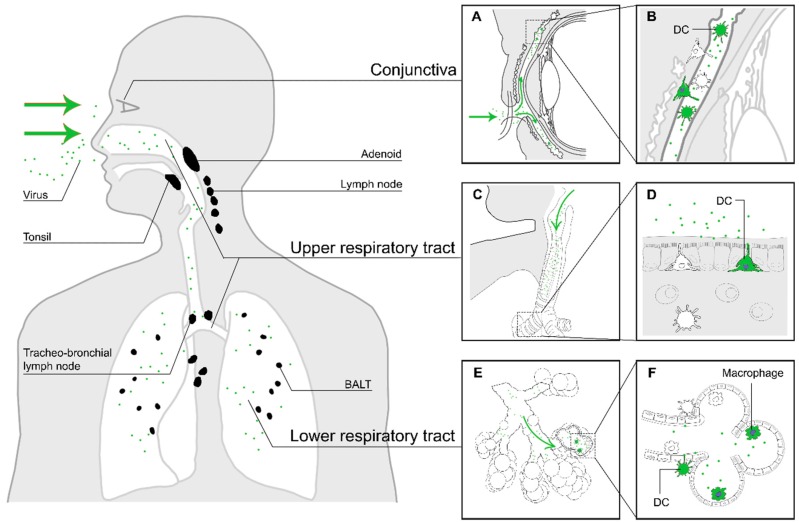
The first stage of MV infection: entry of MV into a susceptible host. The virus enters the respiratory tract (green arrows in panels (**C**) and (**E**)), where it binds to DC-SIGN^+^ DCs or infects CD150^+^ myeloid or lymphoid cells in the mucocilliary epithelium or the alveolar spaces. Another potential site of entry is through the conjunctiva, which is rich in DCs and CD150^+^ lymphocytes (**A**). Panels on the right show an enlarged illustration of potential entry events. MV particles deposited on the conjunctiva will enter the space between cornea and eyelids ((**A**), green arrows), where they can infect myeloid or lymphoid cells (**B**). MV particles inhaled into the respiratory tract ((**C**) and (**E**), green arrows) can either infect DC-SIGN^+^ dendritic cells in the upper respiratory tract, with dendrites protruding into the respiratory mucosa (**D**), or dendritic cells or macrophages in the alveolar lumina of the lower respiratory tract (**F**). The infected immune cells subsequently migrate to nearby tertiary lymphoid tissues and draining lymph nodes (black).

In vivo studies with NHPs experimentally infected with rMV expressing enhanced green fluorescent protein (EGFP) identified CD11c^+^ myeloid cells, most likely alveolar macrophages and DCs, in the lungs and respiratory submucosa as potential early target cells [8,22,23]. Two mechanisms of MV entry were proposed based on these findings: infection of CD150^+^ cells in the alveolar spaces or binding to dendrites of DC-SIGN^+^ submucosal DCs in the lumen of the respiratory tract, followed by migration to tertiary lymphoid tissues, such as the bronchus-associated lymphoid tissue [24], and draining lymph nodes [8,25,26], where the infection is subsequently amplified by massive replication in abundantly present CD150^+^ B- and T-cells [8,27]. These potential routes of entry are illustrated in Figure 1.

Another possible, but probably less important, route of MV entry is through MV infection of myeloid or lymphoid cells in the conjunctiva. The lamina propria of the conjunctiva is rich in DCs, Langerhans cells, macrophages, CD4^+^ and CD8^+^ T-cells and B-cells, providing a suitable site of replication for the virus [28]. This infection and the ensuing MV-specific immune response may subsequently lead to prodromal conjunctivitis [29]. In addition to the conjunctiva, MV has been shown to infect human corneal rim epithelial cells ex vivo [30]. It has been reported that eye protection during contact with measles patients can reduce the risk of contracting infection by MV [31].

Invasion of the respiratory tract by bacteria or other pathogens causing damage to the epithelial layer could be advantageous for MV entry. MV inoculated onto the apical side of well-differentiated ciliated bronchial epithelial cell cultures did not result in infection. However, wounding of the human bronchial epithelial cell in vitro resulted in numerous foci of infection along the lines of the wound, possibly due to the disruption of the tight junctions at cell-to-cell contacts and the subsequent exposure of nectin-4 as a cellular receptor [30,32]. It is possible that MV can infect the respiratory tract through similar epithelial disruption in vivo, either as a result of infection or mechanical damage [30,33]. However, the highly efficient transmission of MV from infected to naive individuals and the susceptibility of every measles-naive human to MV infection suggests that infectious predisposition is not a requirement for efficient MV entry.

## 3. Dissemination

Primary (bone marrow and thymus), secondary (spleen, tonsils, lymph nodes) and tertiary (e.g., bronchus-associated lymphoid tissue (BALT)) lymphoid tissues are rich in CD150^+^ lymphocytes and are major sites of MV replication in vivo [8,25,27,34,35,36,37]. Analysis of lymphoid tissues of experimentally infected NHPs showed prominent MV replication in B-cell follicles [8,25]. Multinucleated giant cells or syncytia, known as Warthin-Finkeldey cells, were especially observed in lymphoid tissues in the upper respiratory tract [25,38,39,40] and consisted of fused B-cells [8,27]. In addition to B-cells, widespread MV infection of both CD4^+^ and CD8^+^ CD150^+^ memory T-cells was observed in these tissues.

**Figure 2:**
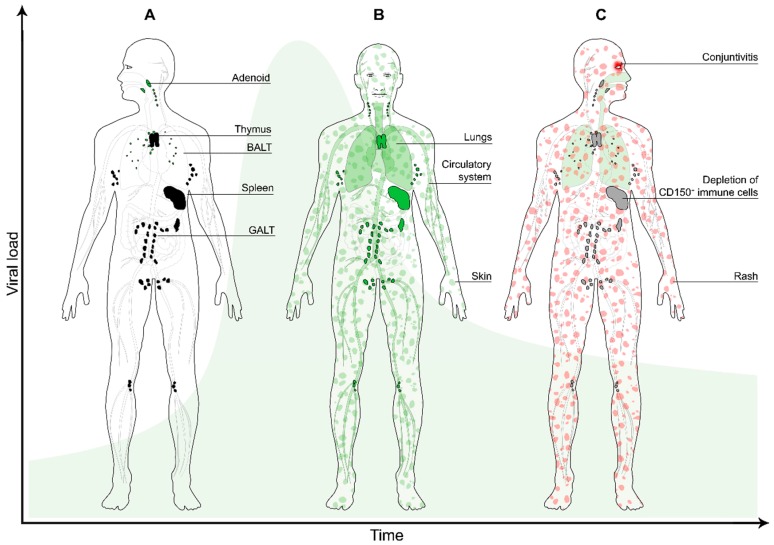
The second stage of MV infection: systemic dissemination. (**A**) The MV-infected myeloid cells migrate to the draining lymph nodes (black), where they transmit the virus to CD150^+^ lymphocytes (predominantly B-cells and memory CD4^+^ and CD8^+^ T-cells); (**B**) during viremia infected cells enter the circulation and migrate systemically to various organs and tissues (green), where the infection is further amplified. Infection of skin-resident immune cells results in virus transmission to nectin-4^+^ epithelial cells (green patches); (**C**) a few days later, depletion of immune cells in lymphoid organs and tissues results in transient immune suppression (grey). MV-specific T-cells infiltrate the skin where they clear the infected cells, which results in the typical measles skin rash (red patches). The green bell-shaped curve in the background represents the viral load over time.

Viral dissemination is predominantly mediated by cell-to-cell transmission of virus [32,41,42]. In MV-infected NHPs, infected cells in peripheral tissues were mostly interconnected by dendrites. Widespread infection of lymphoid tissues is followed by infection of lymphocytes and DCs in the skin and the epithelial submucosa (Figure 2A). Here, infected lymphocytes or DCs transmit the virus to the neighbouring nectin-4^+^ epithelial cells [30,43,44] or keratinocytes. Nectins can form both homodimers and heterodimers at cell-to-cell junctions, but the heterodimer interactions have been shown to be more stable. The disruption of nectin-4 and nectin-1 heterodimers by MV has been suggested to facilitate viral spread [45].

MV spreads systemically to other organs and tissues, such as the gastrointestinal tract, kidney, liver and skin through infected circulating CD150^+^ immune cells (Figure 2B), and, in some rare cases, infects endothelial cells, neurons, astrocytes and oligodendrocytes in vivo [3,46]. MV infection stimulates the expression and activation of the leukocyte integrins lymphocyte function associated antigen-1 and very late activation antigen-4 [47]. These molecules allow adherence of infected migrating cells to the endothelial cells and subsequent trans-migration into the tissues [46,47]. Infection of endothelial cells with MV in vitro stimulates the production of colony-stimulating factor and thus increases the adhesion of granulocytes to infected epithelial cells [48]. MV antigens were found in the capillary endothelium of lymph nodes and thymus in patients who died from the infection [49].

MV can also infect permissive cells through receptor-independent mechanisms [50], although these mechanisms are much less efficient than receptor-mediated entry. One of the possible mechanisms is through an in-cell infection. This mechanism has been identified in allowing Epstein-Barr virus (EBV) spread from infected B-cells to epithelial cells by internalisation of the EBV-infected B-cells into carcinoma cells, resulting in activation and transfer of the virus to the carcinoma cells in vitro and in vivo [51]. It is tempting to speculate that MV-infected lymphocytes can also be internalised by receptor-negative cells, leading to infection.

Clinical measles starts with the emergence of Koplik’s spots on the buccal mucosa and culminates a few days later in the appearance of the maculopapular skin rash (Figure 2C) [3]. Histological examinations showed that the characteristics of the Koplik’s spots were similar to those of the skin rash and they may contain syncytia [52]. The rash can potentially be explained by infection of the dermal endothelial cells and keratinocytes, which are subsequently cleared by the virus-specific host cellular immune response [3]. Several studies reported that viral antigens were found in the corneal layer, spongiotic epidermal keratinocytes and even more in the dermal papillary layer [15]. The skin lesions were characterised with spongiosis, cell necrosis and mononuclear cell infiltration of the epidermal keratinocytes [14,15]. The crucial role of the host immune response in the pathogenesis of the skin rash is illustrated by the fact that immunocompromised patients often do not develop skin rash following MV infection, although the course of a MV infection in these patients is typically severe and can be lethal [53].

Although most measles cases resolve without complications, the virus can remain persistent and infect the CNS on rare occasions. One of the neurologic complications, known as the ADEM, is immune-mediated and has a higher incidence and severity than the other complications (~1:1000). Although the induction of this autoimmune response is poorly understood, “molecular mimicry” based on structural similarities between MV proteins and myelin has been suggested as a pathogenic mechanism [54,55]. The disease is hallmarked by demyelination, which results in ataxia, motor and sensory loss and mental status changes [56] and can result in death.

A second neurologic complication stemming from systemic MV infection is MIBE. The risk of developing MIBE increases when the MV infection occurs in young infants or immunocompromised individuals, who are unable to clear the infection. The symptoms of MIBE often include mental status changes, focal seizures and occasionally visual or hearing loss within one year of acute measles infection or live-virus vaccination [57,58]. The disease progresses rapidly to coma and death in the majority of patients [54].

A third and very rare neurological complication of measles is SSPE. Symptoms develop several years after a normal episode of measles and usually start with a decline of school performance and a slight change of behaviour, progressively followed by myoclonic seizures, ataxia and death within one to three years [25,59]. SSPE is exclusively associated with infections with wild-type MV, and has never been observed in association with genotype A vaccine viruses. Where the virus persists and how it spreads in the CNS remains unknown. It has been suggested that the virus spreads from one neuron to the other through interconnecting processes in vitro and in vivo, without the release of infectious particles [60,61,62]. This infection may rely on membrane fusion between infected and uninfected neurons, allowing trans-synaptic transmission of ribonucleoprotein (RNP) [25,63,64]. The RNP consists of genomic viral RNA encapsidated with the viral nucleoprotein and associated with the viral polymerase, and is the minimal unit of infection [3]. MV-positive oligodendrocytes and astrocytes were also found in the white matter of SSPE cases. The virus may spread from one glial cell to another via interconnected processes [25].

It is still unclear how MV enters the CNS, however in recent years it has become apparent that the blood-brain-barrier allows entry of lymphocytes into the brain [65,66]. Moreover, it has been shown that the brain even contains lymphatic vessels [67]. Therefore, infected lymphocytes circulating in peripheral blood during viremia could carry the virus into the CNS, where the virus could be transmitted via a yet unknown cellular entry receptor or receptor-independent entry mechanisms.

## 4. Transmission

The basic reproductive number (R_0_) reflects the average number of secondary cases that would arise when an infectious agent is introduced into a completely susceptible population [68]. MV is released into the air as cell-free or cell-associated virus particles, predominantly by coughing [9,10,69]. The virus is highly infectious: the estimated R_0_ is 12 to 18 [68]. In specific cases individual patients have been reported to more than 200 new patients [70], often referred to as “superspreading” events [71].

**Figure 3:**
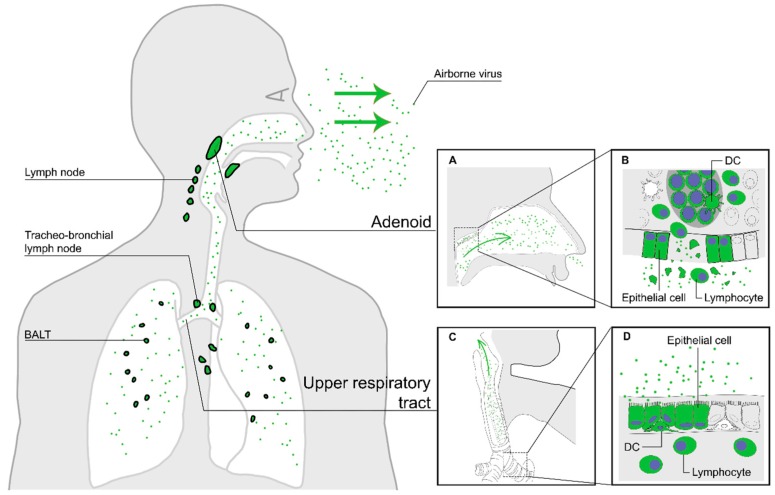
The third stage of MV infection: transmission of new MV particles via the air. Nectin-4^+^ epithelial cells in the upper and lower respiratory tract epithelium produce new virus particles and release them into the mucus lining the lumen of the respiratory tract (green arrows in panels (**A**) and (**C**)). Epithelial damage in infected lymphoid tissues, such as the tonsils (**A**), releases virus particles produced by lymphocytes into the upper respiratory tract (**B**). Epithelial damage in the lower respiratory tract induces cough (panels (**C**) and (**D**)), enhancing the discharge of aerosols containing MV particles.

The high infectivity of MV can be attributed to three crucial transmission properties. First, measles patients must efficiently shed MV. Tracheo-bronchial epithelial cells have been reported to be susceptible to MV infection [10,25,43,44], associated with epithelial damage in the bronchi and bronchioles [27,43]. Whereas epithelial cells are infected from the basolateral side, budding occurs exclusively at the apical cell surface due to sorting signals in the viral glycoproteins. Whilst MV particles produced in the lymphoid tissues can rapidly bind to neighbouring CD150^+^ cells that are highly abundant in the environment, MV particles produced by the respiratory epithelial cells will be shed into the mucus lining the lumen of the respiratory tract where cells expressing MV receptors are scarce. Hence, virions remain in the mucus as cell-free particles, and are moved to the upper respiratory tract (URT) by the mucocilliary escalator [43] and discharged into the environment by coughing. MV can be transmitted by large respiratory droplets (by direct contact) or in small aerosols transported through the air over long distances [72]. The release of new MV particles from the host into the air is illustrated in Figure 3.

Second, the virus must remain infectious until it reaches a new host. Large droplets may increase the stability of cell-bound MV particles or cell debris that are expelled from the body, allowing the virus to survive long enough until it comes into contact with the eyes, nose or mouth of a susceptible person. Alternatively, cell-free virions transmitted airborne as small aerosols through a turbulent airflow may survive in air for at least one hour, as demonstrated during the outbreaks of measles in a paediatric practice in 1981 and at an International Special Olympics Games in 1995 [72,73]. One of the factors that influence survival of MV in the air is relative humidity: in aerosols, the virus is most stable below 40% or above 80% [74].

The last vital transmission property concerns the infectious dose of the virus. In NHPs, a single 50% tissue culture infectious dose was shown to be sufficient to establish a productive infection associated with systemic dissemination [75]. However, measles patients shed large amounts of virus, resulting in transmission of numerous infectious units. The combination of large inoculum and low infectious dose may increase the chance of rapid deposition of virus particles in the respiratory tract of the next host, especially in a crowded and poorly ventilated environment [73].

## 5. Immune Suppression

MV infection results in a transient and profound immune suppression, which leads to increased susceptibility to opportunistic infections and increased childhood mortality [4]. The virus efficiently replicates in lymphoid tissues. Tertiary lymphoid tissues, such as BALT and gut associated lymphoid tissues (GALT), can be induced by bacterial or viral infection that leads to the accumulation and proliferation of lymphocytes and the formation of germinal centres. CD11c^+^ DCs and follicular DCs are present within to maintain the structure of these tissues [76,77]. The presence and interaction of CD150^+^ lymphocytes and DC-SIGN^+^ DCs in these tissues consequently makes them the perfect site for MV infection and amplification [8,27]. Since BALT and GALT are known to enhance protective immunity against mucosal pathogens, obliteration of these lymphoid tissues that are present in major entry portals for opportunistic infections (the airways and gut) can facilitate infiltration of the mucosa by previously encountered viruses or bacteria.

MV infection leads to lymphopenia during its acute phase, in which the number of T- and B-cells, both circulating and lymphoid tissue homing, decreases extensively (Figure 2C) [27,78]. Peak numbers of MV-infected cells in lymphoid tissues of experimentally infected NHPs coincide with the peak of viremia, rapidly followed by B-cell exhaustion in the germinal centres [27], as previously also reported in humans [38]. The infection induces an expansive effector phase, leading to the clearance of MV-infected cells by cytotoxic T-cells [79] and subsequently a lifelong measles-specific immune response [80]. Following viral clearance, the number of lymphocytes returns to normal within approximately one week. However, while the lymphopenia lasts for a week, the immune suppression may last variably from several weeks to up to more than two years [4]. This led to the initial dismissal of the role of immune cell depletion in causing measles-induced immune suppression [81]. Instead, functional impairment of the immune cells has often been proposed to explain the mechanism of the immune suppression. However, there is limited evidence that this is the case and it has proven difficult to identify a cell surface receptor that mediates suppression of proliferation in immune cells. Reduced proliferative responsiveness of peripheral blood lymphocytes to antigenic or mitogenic stimulation has also been suggested as a mechanism of measles immune suppression. Although this impairment is indeed detected in vitro, measles is associated with dramatic levels of lymphoproliferation in vivo [27]. Other mechanisms have been proposed to explain the nature of the measles-induced immune suppression, such as altered cytokine profiles [82,83,84,85] or inhibited haematopoiesis [86,87], but none of these fit with the measles paradox: Prolonged increased susceptibility to infectious disease and coinciding induction of strong MV-specific immune responses.

Based on observations in experimentally infected NHPs we proposed an alternative model explaining measles immune suppression, based on the preferential infection and subsequent immune-mediated depletion of CD150^+^ memory T- and B-cells, resulting in “immune amnesia” [27,88]. The loss of memory lymphocytes is masked by a massive expansion of new MV-specific and bystander lymphocytes, explaining the short duration of lymphopenia and yet the long duration of immune suppression. This finding thus revives the importance of immune cell depletion as a key mechanism for measles-associated immune suppression.

**Figure 4:**
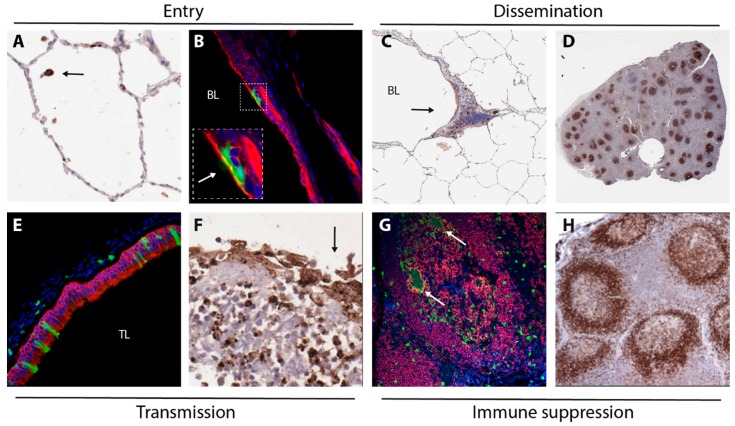
Images collected from experimentally infected NHPs, illustration mechanisms underlying MV entry (**A**,**B**), dissemination (**C**,**D**), transmission (**E**,**F**) and immune suppression (**G**,**H**). MV-infected cells were detected by immunohistochemical staining (**A**,**C**,**D**,**F**) or by immunofluorescent double-staining (**B**,**E**,**G**). (**A**) infection of a single cell (arrow, likely an alveolar macrophage) in the alveolar lumen 3 DPI; (**B**) infection of epithelial cells in the trachea 5 DPI (arrow in insert points at green cilia), green = GFP, red = cytokeratin, blue = DAPI; (**C**) infection of myeloid and lymphoid cells in BALT (arrow) 4 DPI, BL = bronchial lumen; (**D**) low-magnification image of a lymph node 9 DPI, with many B-cell follicles containing large concentrations of MV-infected lymphocytes; (**E**) MV-infected epithelial cells in the trachea 9 DPI (green = GFP, red = cytokeratin, blue = DAPI, TL = tracheal lumen); (**F**) Disruption of the epithelium (arrow) of an adenoid containing many MV-infected lymphocytes 9 DPI; (**G**) MV-infected B-lymphocytes (including Warthin-Finkeldey syncytia, arrows) in a B-cell follicle 9 DPI (green = GFP, red = CD20, blue = DAPI); (**H**) follicular exhaustion of B-cell follicles 11 DPI (brown = CD20).

Mechanisms underlying MV entry, dissemination, transmission and immune suppression as discussed in this review are illustrated with images from experimentally infected NHPs in Figure 4.

## 6. Conclusions

Measles has caused a high number of fatalities throughout history. Recombinant viruses expressing fluorescent reporter proteins have given us the means to study and understand the virus and its pathogenesis from a new perspective. However, these advances not only leave some old mysteries concerning measles pathogenesis unexplained, but also give birth to new questions. Since global eradication of measles is planned for the near future, studies on MV tropism and pathogenesis not only remain important, but also become urgent [89].

## Abbreviations

The following abbreviations are used in this manuscript:

MV	measles virus
NHP	non-human primate
CNS	central nervous system
ADEM	acute disseminated encephalomyelitis
MIBE	measles inclusion body encephalitis
SSPE	subacute sclerosing panencephalitis
WHO	World Health Organisation
SLAMF1	signalling lymphocyte activation molecule family member 1
DCs	dendritic cells
HSC	haematopoietic stem cells
rMV	recombinant measles virus
DC-SIGN	dendritic cell-specific intercellular adhesion molecule-3-grabbing non-integrin
EGFP	enhanced green fluorescent protein
HRSV	human respiratory syncytial virus
BALT	bronchus-associated lymphoid tissues
EBV	Epstein-Barr virus
RNP	ribonucleoprotein
URT	upper respiratory tract
GALT	gut-associated lymphoid tissues

## References

[1] De Vries R.D., Duprex W.P., de Swart R.L. (2015). Morbillivirus infections: An introduction. Viruses.

[2] Yanagi Y., Takeda M., Ohno S. (2006). Measles virus: Cellular receptors, tropism and pathogenesis. J. Gen. Virol..

[3] Griffin D.E. (2013). Measles virus. Fields Virology.

[4] Mina M.J., Metcalf C.J., de Swart R.L., Osterhaus A.D., Grenfell B.T. (2015). Long-term measles-induced immunomodulation increases overall childhood infectious disease mortality. Science.

[5] Perry R.T., Murray J.S., Gacic-Dobo M., Dabbagh A., Mulders M.N., Strebel P.M., Okwo-Bele J.M., Rota P.A., Goodson J.L. (2015). Progress towards regional measles elimination, worldwide, 2000–2014. Wkly. Epidemiol. Rec..

[6] Tatsuo H., Ono N., Tanaka K., Yanagi Y. (2000). SLAM (CDw150) is a cellular receptor for measles virus. Nature.

[7] Schwartzberg P.L., Mueller K.L., Qi H., Cannons J.L. (2009). SLAM receptors and SAP influence lymphocyte interactions, development and function. Nat. Rev. Immunol..

[8] De Swart R.L., Ludlow M., de Witte L., Yanagi Y., van Amerongen G., McQuaid S., Yuksel S., Geijtenbeek T.B., Duprex W.P., Osterhaus A.D. (2007). Predominant infection of CD150^+^ lymphocytes and dendritic cells during measles virus infection of macaques. PLoS Pathog..

[9] Noyce R.S., Bondre D.G., Ha M.N., Lin L.T., Sisson G., Tsao M.S., Richardson C.D. (2011). Tumor cell marker PVRL4 (nectin 4) is an epithelial cell receptor for measles virus. PLoS Pathog..

[10] Muhlebach M.D., Mateo M., Sinn P.L., Prufer S., Uhlig K.M., Leonard V.H., Navaratnarajah C.K., Frenzke M., Wong X.X., Sawatsky B. (2011). Adherens junction protein nectin-4 is the epithelial receptor for measles virus. Nature.

[11] Mollo M.R., Antonini D., Mitchell K., Fortugno P., Costanzo A., Dixon J., Brancati F., Missero C. (2015). p63-dependent and independent mechanisms of nectin-1 and nectin-4 regulation in the epidermis. Exp. Dermatol.

[12] Brancati F., Fortugno P., Bottillo I., Lopez M., Josselin E., Boudghene-Stambouli O., Agolini E., Bernardini L., Bellacchio E., Iannicelli M. (2010). Mutations in PVRL4, encoding cell adhesion molecule nectin-4, cause ectodermal dysplasia-syndactyly syndrome. Am. J. Hum. Genet..

[13] Abdullah H., Brankin B., Brady C., Cosby S.L. (2013). Wild-type measles virus infection upregulates poliovirus receptor-related 4 and causes apoptosis in brain endothelial cells by induction of tumor necrosis factor-related apoptosis-inducing ligand. J. Neuropathol. Exp. Neurol..

[14] Kimura A., Tosaka K., Nakao T. (1975). Measles rash. I. Light and electron microscopic study of skin eruptions. Arch. Virol..

[15] Takahashi H., Umino Y., Sato T.A., Kohama T., Ikeda Y., Iijima M., Fujisawa R. (1996). Detection and comparison of viral antigens in measles and rubella rashes. Clin. Infect. Dis..

[16] Dörig R.E., Marcil A., Chopra A., Richardson C.D. (1993). The human CD46 molecule is a receptor for measles virus (Edmonston strain). Cell.

[17] Buckland R., Wild T.F. (1997). Is CD46 the cellular receptor for measles virus?. Virus Res..

[18] De Witte L., de Vries R.D., van der Vlist M., Yuksel S., Litjens M., de Swart R.L., Geijtenbeek T.B. (2008). DC-SIGN and CD150 have distinct roles in transmission of measles virus from dendritic cells to T-lymphocytes. PLoS Pathog..

[19] Van der Vlist M., de Witte L., de Vries R.D., Litjens M., de Jong M.A., Fluitsma D., de Swart R.L., Geijtenbeek T.B. (2011). Human Langerhans cells capture measles virus through Langerin and present viral antigens to CD4^+^ T cells but are incapable of cross-presentation. Eur. J. Immunol..

[20] Leonard V.H., Sinn P.L., Hodge G., Miest T., Devaux P., Oezguen N., Braun W., McCray P.B., McChesney M.B., Cattaneo R. (2008). Measles virus blind to its epithelial cell receptor remains virulent in rhesus monkeys but cannot cross the airway epithelium and is not shed. J. Clin. Investig..

[21] Leonard V.H., Hodge G., Reyes-Del Valle J., McChesney M.B., Cattaneo R. (2010). Measles virus selectively blind to signaling lymphocytic activation molecule (SLAM; CD150) is attenuated and induces strong adaptive immune responses in rhesus monkeys. J. Virol..

[22] De Vries R.D., Lemon K., Ludlow M., McQuaid S., Yuksel S., van Amerongen G., Rennick L.J., Rima B.K., Osterhaus A.D., de Swart R.L. (2010). In vivo tropism of attenuated and pathogenic measles virus expressing green fluorescent protein in macaques. J. Virol..

[23] Mesman A.W., de Vries R.D., McQuaid S., Duprex W.P., de Swart R.L., Geijtenbeek T.B.H. (2012). A prominent role for DC-SIGN^+^ dendritic cells in initiation and dissemination of measles virus infection in non-human primates. PLoS ONE.

[24] Lemon K., de Vries R.D., Mesman A.W., McQuaid S., van Amerongen G., Yuksel S., Ludlow M., Rennick L.J., Kuiken T., Rima B.K. (2011). Early target cells of measles virus after aerosol infection of non-human primates. PLoS Pathog..

[25] Ludlow M., McQuaid S., Milner D., de Swart R.L., Duprex W.P. (2015). Pathological consequences of systemic measles virus infection. J. Pathol..

[26] De Vries R.D., Mesman A.W., Geijtenbeek T.B., Duprex W.P., de Swart R.L. (2012). The pathogenesis of measles. Curr. Opin. Virol..

[27] De Vries R.D., McQuaid S., van Amerongen G., Yuksel S., Verburgh R.J., Osterhaus A.D., Duprex W.P., de Swart R.L. (2012). Measles immune suppression: Lessons from the macaque model. PLoS Pathog..

[28] Seo K.Y., Han S.J., Cha H.R., Seo S.U., Song J.H., Chung S.H., Kweon M.N. (2010). Eye mucosa: An efficient vaccine delivery route for inducing protective immunity. J. Immunol..

[29] Papp K. (1954). Contagion des virus a travers une conjonctive intacte; rougeole, oreillons, rubéole. Rev. Immunol. Ther. Antimicrob..

[30] Ludlow M., Rennick L.J., Sarlang S., Skibinski G., McQuaid S., Moore T., de Swart R.L., Duprex W.P. (2010). Wild-type measles virus infection of primary epithelial cells occurs via the basolateral surface without syncytium formation or release of infectious virus. J. Gen. Virol..

[31] Papp K. (1956). Expériences prouvant que la voie d’infection de la rougeole est la contamination de la muqueuse conjonctivale. Rev. Immunol. Ther. Antimicrob..

[32] Singh B.K., Hornick A.L., Krishnamurthy S., Locke A.C., Mendoza C.A., Mateo M., Miller-Hunt C.L., Cattaneo R., Sinn P.L. (2015). The nectin-4/afadin protein complex and intercellular membrane pores contribute to rapid spread of measles virus in primary human airway epithelia. J. Virol..

[33] Message S.D., Johnston S.L. (2001). The immunology of virus infection in asthma. Eur. Respir. J..

[34] Sakaguchi M., Yoshikawa Y., Yamanouchi K., Sata T., Nagashima K., Takeda K. (1986). Growth of measles virus in epithelial and lymphoid tissues of cynomolgus monkeys. Microb. Immunol..

[35] Hall W.C., Kovatch R.M., Herman P.H., Fox J.G. (1971). Pathology of measles in rhesus monkeys. Vet. Pathol..

[36] McChesney M.B., Miller C.J., Rota P.A., Zhu Y.D., Antipa L., Lerche N.W., Ahmed R., Bellini W.J. (1997). Experimental measles. I. Pathogenesis in the normal and the immunized host. Virology.

[37] White R.G., Boyd J.F. (1973). The effect of measles on the thymus and other lymphoid tissues. Clin. Exp. Immunol..

[38] Warthin A.S. (1931). Occurence of numerous large giant cells in the tonsils and pharyngeal mucosa in the prodormal stage of measles. Arch. Pathol..

[39] Finkeldey W. (1931). Über Riesenzellbefunde in den Gaumenmandeln, zugleich ein Beitrag zur Histopathologie der Mandelveränderungen im Maserninkubationsstadium. Virchows Arch..

[40] Stryker W.A. (1940). Disseminated giant cell reaction: A possible prodrome of measles. Am. J. Dis. Child..

[41] Koethe S., Avota E., Schneider-Schaulies S. (2012). Measles virus transmission from dendritic cells to T cells: Formation of synapse-like interfaces concentrating viral and cellular components. J. Virol..

[42] Mateo M., Generous A., Sinn P.L., Cattaneo R. (2015). Connections matter—How viruses use cell-cell adhesion components. J. Cell. Sci.

[43] Ludlow M., Lemon K., de Vries R.D., McQuaid S., Millar E.L., van Amerongen G., Yuksel S., Verburgh R.J., Osterhaus A.D., de Swart R.L. (2013). Measles virus infection of epithelial cells in the macaque upper respiratory tract is mediated by subepithelial immune cells. J. Virol..

[44] Frenzke M., Sawatsky B., Wong X.X., Delpeut S., Mateo M., Cattaneo R., von Messling V. (2013). Nectin-4-dependent measles virus spread to the cynomolgus monkey tracheal epithelium: Role of infected immune cells infiltrating the lamina propria. J. Virol..

[45] Mateo M., Navaratnarajah C.K., Willenbring R.C., Maroun J.W., Iankov I., Lopez M., Sinn P.L., Cattaneo R. (2014). Different roles of the three loops forming the adhesive interface of nectin-4 in measles virus binding and cell entry, nectin-4 homodimerization, and heterodimerization with nectin-1. J. Virol..

[46] Ludlow M., Allen I., Schneider-Schaulies J. (2009). Systemic spread of measles virus: Overcoming the epithelial and endothelial barriers. Thromb. Haemost.

[47] Dittmar S., Harms H., Runkler N., Maisner A., Kim K.S., Schneider-Schaulies J. (2008). Measles virus-induced block of transendothelial migration of T lymphocytes and infection-mediated virus spread across endothelial cell barriers. J. Virol..

[48] Friedman H.M. (1989). Infection of endothelial cells by common human viruses. Rev. Infect. Dis..

[49] Moench T.R., Griffin D.E., Obriecht C.R., Vaisberg A.J., Johnson R.T. (1988). Acute measles in patients with and without neurological involvement: Distribution of measles virus antigen and RNA. J. Infect. Dis..

[50] Hashimoto K., Ono N., Tatsuo H., Minagawa H., Takeda M., Takeuchi K., Yanagi Y. (2002). SLAM (CD150)-independent measles virus entry as revealed by recombinant virus expressing green fluorescent protein. J. Virol..

[51] Lee Y., Overholtzer M. (2015). In-cell infection: Bringing uninvited guests. Cell. Res..

[52] Suringa D.W., Bank L.J., Ackerman A.B. (1970). Role of measles virus in skin lesions and Koplik‘s spots. N. Engl. J. Med..

[53] De Swart R.L., Wertheim-van Dillen P.M., van Binnendijk R.S., Muller C.P., Frenkel J., Osterhaus A.D. (2000). Measles in a Dutch hospital introduced by an immuno-compromised infant from Indonesia infected with a new virus genotype. Lancet.

[54] Griffin D.E. (2014). Measles virus and the nervous system. Handb. Clin. Neurol..

[55] Fujinami R.S., Oldstone M.B., Wroblewska Z., Frankel M.E., Koprowski H. (1983). Molecular mimicry in virus infection: Crossreaction of measles virus phosphoprotein or of herpes simplex virus protein with human intermediate filaments. Proc. Natl. Acad. Sci. USA.

[56] Buchanan R., Bonthius D.J. (2012). Measles virus and associated central nervous system sequelae. Semin. Pediatr. Neurol..

[57] Freeman A.F., Jacobsohn D.A., Shulman S.T., Bellini W.J., Jaggi P., de Leon G., Keating G.F., Kim F., Pachman L.M., Kletzel M. (2004). A new complication of stem cell transplantation: Measles inclusion body encephalitis. Pediatrics.

[58] Bitnun A., Shannon P., Durward A., Rota P.A., Bellini W.J., Graham C., Wang E., Ford-Jones E.L., Cox P., Becker L. (1999). Measles inclusion-body encephalitis caused by the vaccine strain of measles virus. Clin. Infect. Dis..

[59] Perry R.T., Halsey N.A. (2004). The clinical significance of measles: A review. J. Infect. Dis..

[60] Ehrengruber M.U., Ehler E., Billeter M.A., Naim H.Y. (2002). Measles virus spreads in rat hippocampal neurons by cell-to-cell contact and in a polarized fashion. J. Virol..

[61] Duprex W.P., McQuaid S., Roscic-Mrkic B., Cattaneo R., McCallister C., Rima B.K. (2002). In vitro and in vivo infection of neural cells by a recombinant measles virus expressing enchanced green fluorescent protein. J. Virol..

[62] Lawrence D.M.P., Patterson C.E., Gales T.L., D‘orazio J.L., Vaughn M.M., Rall G.F. (2000). Measles virus spread between neurons requires cell contact but not CD46 expression, syncytium formation, or extracellular virus production. J. Virol..

[63] Makhortova N.R., Askovich P., Patterson C.E., Gechman L.A., Gerard N.P., Rall G.F. (2007). Neurokinin-1 enables measles virus trans-synaptic spread in neurons. Virology.

[64] Watanabe S., Shirogane Y., Suzuki S.O., Ikegame S., Koga R., Yanagi Y. (2013). Mutant fusion proteins with enhanced fusion activity promote measles virus spread in human neuronal cells and brains of suckling hamsters. J. Virol..

[65] Bechmann I., Galea I., Perry V.H. (2007). What is the blood-brain barrier (not)?. Trends Immunol..

[66] Ransohoff R.M., Engelhardt B. (2012). The anatomical and cellular basis of immune surveillance in the central nervous system. Nat. Rev. Immunol..

[67] Louveau A., Smirnov I., Keyes T.J., Eccles J.D., Rouhani S.J., Peske J.D., Derecki N.C., Castle D., Mandell J.W., Lee K.S. (2015). Structural and functional features of central nervous system lymphatic vessels. Nature.

[68] Moss W.J., Griffin D.E. (2006). Global measles elimination. Nat. Rev. Microb..

[69] Racaniello V. (2011). Virology. An exit strategy for measles virus. Science.

[70] Christensen P.E., Schmidt H., Jensen O., Bang H.O., Andersen V., Jordal B. (1953). An epidemic of measles in Southern Greenland, 1951. I. Measles in virgin soil. Acta Med. Scand..

[71] Lloyd-Smith J.O., Schreiber S.J., Kopp P.E., Getz W.M. (2005). Superspreading and the effect of individual variation on disease emergence. Nature.

[72] Bloch A.B., Orenstein W.A., Ewing W.M., Spain W.H., Mallison G.F., Herrmann K.L., Hinman A.R. (1985). Measles outbreak in a pediatric practice: Airborne transmission in an office setting. Pediatrics.

[73] Ehresmann K.R., Hedberg C.W., Grimm M.B., Norton C.A., Macdonald K.L., Osterholm M.T. (1995). An outbreak of measles at an international sporting event with airborne transmission in a domed stadium. J. Infect. Dis..

[74] De Jong J.G., Winkler K.C. (1964). Survival of measles virus in air. Nature.

[75] Van Binnendijk R.S., van der Heijden R.W., van Amerongen G., UytdeHaag F.G., Osterhaus A.D. (1994). Viral replication and development of specific immunity in macaques after infection with different measles virus strains. J. Infect. Dis..

[76] Foo S.Y., Phipps S. (2010). Regulation of inducible BALT formation and contribution to immunity and pathology. Mucosal Immunol..

[77] GeurtsvanKessel C.H., Willart M.A., Bergen I.M., van Rijt L.S., Muskens F., Elewaut D., Osterhaus A.D., Hendriks R., Rimmelzwaan G.F., Lambrecht B.N. (2009). Dendritic cells are crucial for maintenance of tertiary lymphoid structures in the lung of influenza virus-infected mice. J. Exp. Med..

[78] Ryon J.J., Moss W.J., Monze M., Griffin D.E. (2002). Functional and phenotypic changes in circulating lymphocytes from hospitalized zambian children with measles. Clin. Diagn. Lab. Immunol..

[79] De Vries R.D., Yuksel S., Osterhaus A.D., de Swart R.L. (2010). Specific CD8^+^ T-lymphocytes control dissemination of measles virus. Eur. J. Immunol..

[80] Griffin D.E., Ward B.J., Jauregui E., Johnson R.T., Vaisberg A. (1989). Immune activation in measles. N. Engl. J. Med..

[81] Griffin D.E. (2010). Measles virus-induced suppression of immune responses. Immunol. Rev..

[82] Ward B.J., Griffin D.E. (1993). Changes in cytokine production after measles virus vaccination: Predominant production of IL-4 suggests induction of a Th2 response. Clin. Immunol. Immunopathol..

[83] Polack F.P., Hoffman S.J., Moss W.J., Griffin D.E. (2002). Altered synthesis of interleukin-12 and type 1 and type 2 cytokinesin rhesus macaques during measles and atypical measles. J. Infect. Dis..

[84] Atabani S.F., Byrnes A.A., Jaye A., Kidd I.M., Magnusen A.F., Whittle H., Karp C.L. (2001). Natural measles causes prolonged suppression of interleukin-12 production. J. Infect. Dis..

[85] Karp C.L., Wysocka M., Wahl L.M., Ahearn J.M., Cuomo P.J., Sherry B., Trinchieri G., Griffin D.E. (1996). Mechanism of suppression of cell-mediated immunity by measles virus. Science.

[86] Manchester M., Smith K.A., Eto D.S., Perkin H.B., Torbett B.E. (2002). Targeting and hematopoietic suppression of human CD34^+^ cells by measles virus. J. Virol..

[87] Boussaad I., Varagnolo L., Hornich V., Rieger L., Krockenberger M., Stuehmer T., Kranzfelder D., Mueller A.M., Schneider-Schaulies S. (2011). Wild-type measles virus interferes with short-term engraftment of human CD34^+^ hematopoietic progenitor cells. J. Virol..

[88] De Vries R.D., de Swart R.L. (2014). Measles immune suppression: Functional impairment or numbers game?. PLoS Pathog..

[89] Nambulli S., Sharp C.R., Acciardo A.S., Drexler J.F., Duprex W.P. (2016). Mapping the evolutionary trajectories of morbilliviruses: What, where and whither. Curr. Opin. Virol..

